# Kids' motives of sport participation and the image of handball–a qualitative approach to increasing the number of child members

**DOI:** 10.3389/fspor.2026.1803270

**Published:** 2026-05-07

**Authors:** Stefan König, Elke Uhl, Alisa Braun

**Affiliations:** Department of Sport Science, Weingarten University of Education, Weingarten, Germany

**Keywords:** children's handball, German Handball Federation, membership, motive-image-fit approach, sustainable development

## Abstract

Membership in the German Handball Federation (DHB) has been declining across all age groups for around 10 years, at least until 2022. Specifically, this trend is reflected in a loss of almost 30% of teams in the younger age groups and even in one third in the age groups of juveniles. For handball in Germany, this is a worrying development. It raises questions about both reasons for this development and how a leading sport association should tackle the problem to ensure a sustainable future of its own sport. These demands were explored in a cooperative project between Weingarten University of Education and the German Sport University Cologne in collaboration with the German Handball Federation. Our common theoretical basis was the motive-image-fit approach. It assumes that children and young people only turn to handball or remain loyal to it if the subjective perception of this sport (image) and the motive structures of the children and young people match. Against this backdrop, two research questions took center stage aiming at children's motives for playing handball and identifying their image of handball. To answer these research questions, an exploratory sequential mixed methods study (QUAL ⇨ QUAN) has been implemented, the first part of which we report on here. The Weingarten research group conducted 35 semi-standardized interviews with 8–12-year-old children. The analysis was carried out using the qualitative content analysis method and MAXQDA 24 software, which led to the development of a deductive-inductive category system. On the basis of 35 interviews we found out that handball is an inspiring, success-oriented and community-oriented sport for many children. In addition, it promotes self-efficacy and team spirit. While the sport is perceived as dynamic by active players, children who do not actively play handball describe it as challenging due to a complex set of rules or the pressure to perform. Thus, our findings contribute to a differentiated view of the motive-image fit and can also contribute to the sustainable development of handball as a sport.

## Introduction

The term sustainability is normally used in the context of ecology, resource management, and environmental protection, and includes the objectives of satisfying the claims and needs of current generations without endangering the opportunities of the future ones. In the context of sports, the concept of sustainability encompasses several completely different issues, ranging from the traditional position of ecological issues to the question of preserving a sport in its existence. Whereas the first idea aims at striking a balance between the demands of our own and contemporary sporting activities, e.g., landscape erosion due to the construction of new sports facilities, and their consequences for the future of our environment, the latter issue is undoubtedly linked to the question of recruiting and retaining members because without people a sport gradually vanishes.

Before this background our article is based on the development of membership and team numbers in the German Handball Federation (DHB) focusing the age groups of children (7–14 years). Between 2009 and 2019, the DHB lost a total of almost 100,000 members. Hereby, children are particularly affected, as this age group accounts for a great deal of the decline (1). This is significant because a comparison with basketball, football, and volleyball does not bring to light a uniform trend: According to DOSB statistics, these sports recorded significantly lower membership losses in this age group between 2009 and 2019, or they even grew, especially after successful international tournaments. Consequently, we must ask ourselves, whether the image of handball (i.e., the picture that children have of this sport) as a more or less traditional sports is one reason for the downward spiral in membership. This automatically brings more differentiated considerations to the fore, such as possible image-promoting and consequently ‘attractive’ characteristics, but also image-damaging and ‘repulsive’ characteristics. In addition, we focused a second key area: children's motives for both participating in sports and playing handball. However, the specific novelty of our study lies in the integration of these two perspectives, the motive-image-fit approach. This objective appears significant from both a theoretical and practical perspective: From a theoretical standpoint, it helps to close a research gap, as research on motivation and participation in sports has predominantly focused on subjective motives, while subjective person-environment fits have been neglected. From a practical perspective, this issue in handball can be applied to other sports. Even though handball is currently grappling with the problem of declining membership, the principle of motivation-image fit could also serve as an important framework for other sports.

From the perspective of the German Handball Federation, these figures and developments as to membership are alarming and they raise the question of what the reasons for this are. Thus, it is our first focus to understand why children and juveniles play and stop playing handball. At the same time, the specific reasons for the loss of young members in handball could lead to conclusions as to why girls and boys in childhood and adolescence generally stay away from handball clubs. Therefore, the article's second focus is on considerations that deal with the sport of handball itself, its characteristics, its degree of organization and its perception from an outside perspective. We believe that if a responsible organization is aware of such characteristics, it has the opportunity to work on the image of its own sport in order to get children excited about the sport and thus attract new members, as well as to retain young girls and boys in the sport and thereby prevent dropouts.

Therefore, the aim of this article is to present the explorative nature of the study mentioned above in which we have analyzed children's motives for taking part in this sport as well as the reasons for ending their career, their image of handball and addressed the following research questions:

(1) What motives are relevant for them to play handball?
(2) What are the main reasons for children to end their handball careers?
(3) What image do children have about handball?

To answer these questions, we first describe the situation within the DHB in comparison to other sport associations in Germany. Second, we draw a theoretical framework encompassing the issue of sport motives, of the image of handball, as well as the fit of these two concepts. We then present a qualitative study having been conducted within the larger cooperative research project MIPHa (Motive-image-fit in handball) conducted by the University of Education Weingarten, Germany, (team König, Braun, & Uhl—Department for Sports Science) and the German Sport University Cologne (team Kleinert, Boss, Hartmann, & Wiesen—Department for Health Psychology). Finally, we draw some conclusions to address the responsible sport associations.

## The situation in Germany

**Figure 1:**
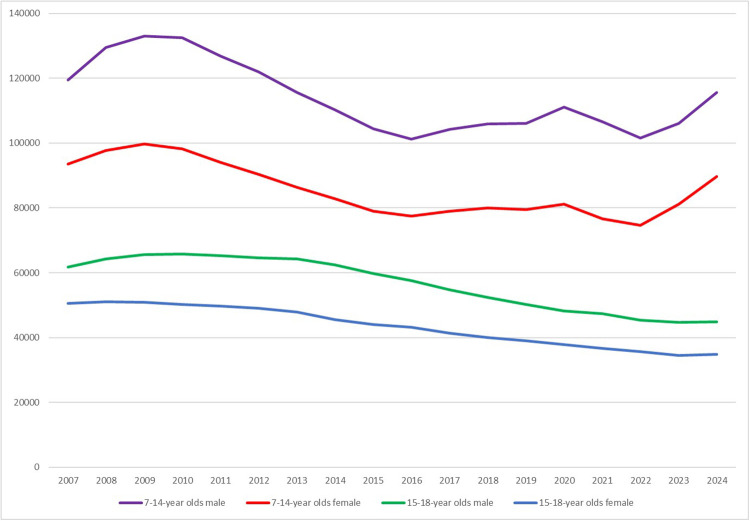
Children's membership development in German handball (based on DHB statistics).

Since winning the 2007 Men's World Championship and after a brief upswing immediately thereafter, membership and team numbers in the German Handball Federation (DHB) have been declining for girls and women as well as for boys and men across all age groups for more than ten years, at least until the year 2022. Children and adolescents aged 7 to 18 are particularly affected in this process, because they account for 75% of the total loss (1). In terms of team numbers, the younger age groups (7–14 years) recorded a loss of just under 30% over the same period. Even if this does not apply equally to all regional handball associations, it has to be stated that it is a rather worrying development for girls' and boys' handball in Germany. Yet, it should also be noted that the trend line has been rising slightly again since the season of 2022/23. This might be due to the fact that both the 2020s have been declared as “the decade of handball in Germany” due to various international tournaments (e.g., women's and men's world championship tournaments in 2025 and 2027), thus the enormous advertising measures are showing initial success, and the DHB has been following a specific strategy for membership recruitment and membership binding for several years. Nevertheless, it is important to consider how this trend can be consolidated and further losses can be avoided in the near future, especially as declines in percentage terms (>25%) can still be observed. Figure 1 gives an overview of the membership developments of children within the DHB, separated by boys and girls.

If we go one step further and compare handball with the other most widespread team sports in Germany at this point (basketball, soccer, volleyball), such a development cannot be observed uniformly. According to the German Olympic Sports Association (DOSB) and the respective national associations' statistics (2), between 2016 and 2025 these sports have recorded significantly lower membership losses in the age groups of 7 to 14 years old, and, in a few cases, even increases can be observed with regard to the last five years: While basketball has been reporting steady membership growth from 2016 to 2025 (trend line) and can report an increase of +79,9% of 7–14 years old children and juveniles, soccer lost the highest absolute number of memberships (approx. 152.000) between 2016 and 2021 (−11,2%); however, the latest figures show a clear increase of ca. 229.500 (+18.9%). Also in volleyball, a sport practiced mainly by 7–14 years old girls, we can note an increase in the last ten years. This growth of 73.6% between 2016 and 2025 must be divided into two time periods, namely a first between 2016 and 2021 with a loss of 10.4% (−5.267 children), and a second since 2021 with an increase of 42.7% (+ 93%). The latter simultaneously represents the highest increase across all our analyses. Table 1 gives an overview of the absolute membership figures of 7 to 14 years old children and juveniles from 2007 to 2025.

**Table 1:** Membership figures of 7 to 14 years old in basketball, soccer, volleyball, and handball (https://cdn.dosb.de/user_upload/www.dosb.de/uber_uns/Bestandserhebung).

Jahr	Basketball	Soccer	Volleyball	Handball
2016	57412	1363487	50842	178660
2017	60813	1321640	50066	183217
2018	62615	1303923	49697	185884
2019	65050	1262443	49576	185583
2020	68615	1266378	49576	192354
2021	62836	1211529	45575	183171
2022	67802	1256323	50596	176050
2023	86003	1336522	60811	187129
2024	103262	1440991	67129	205169
2025	110675	1531753	71080	226029

Summarizing this brief analysis of membership development in the most widespread team sports in Germany, we can state that focusing on the last decade there was a decline in soccer, handball, and volleyball until 2021. It was particularly pronounced in 2020 and 2021 due to the Corona pandemic. Afterwards membership figures increased in all four team sports. However, such a development represents a situation of competition between different sports combined with short-term reversals. Thus, we consider it urgently necessary that the DHB should have more knowledge referring to the image of handball and to children's motives for sport participation to develop and implement a successful recruitment strategy for the sake of the sustainability of its sport.

## Theoretical framework

In the previous section, we were able to show that the DHB will have to struggle with the problem of recruiting and retaining members in the upcoming years, especially because he is in competition with other sport associations. This observation is particularly relevant for adolescents from 7 to 14, even though initial improvements are becoming apparent in this age group. However, a deeper understanding of this issue requires a theoretical examination of these specific phenomena, focusing on concepts of participation in sports clubs, the image of sports, and the motivational state of children and young people to actively participate in sports.

**Figure 2:**
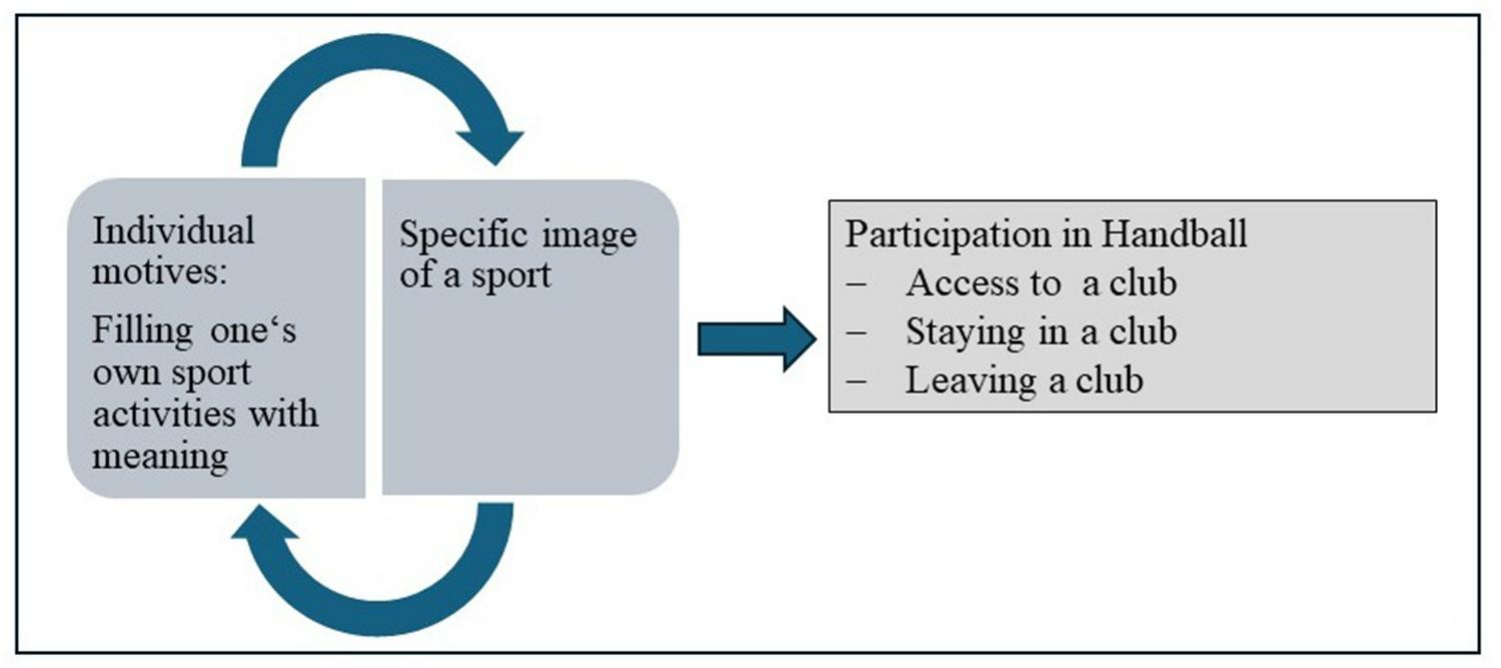
Concept of the motive-image-fit as a reason for participating in handball.

In our research project we have worked with a theoretical concept of participation in clubs as a process of action encompassing three different phases, i.e., access, staying, and leaving, and of being realized within a person-environment-interaction. As a consequence, the notion of person-environment-interaction requires a topic-specific concretization. This has been realized with the idea of the motive-image fit. Motive-image-fit means that access to handball and thus to a sports club is likely, if the subjective image of the context of handball, or the entirety of all perceptions, ideas, and evaluations of the sport of handball, suggests that it satisfies individual needs or fulfills individual meanings, purposes, or goals. In other words, there is a fit between the individual motive for sports participation and the subjective image of the sport of handball. At the same time, the context of handball or the situational anticipations associated with it are attributed with the satisfaction of needs or a sense of purpose, or the fulfillment of goals and objectives is assumed [cf. also contextual offers or possibilities (3)]. This process of matching personal motivational structures with anticipated or perceived situations is a frequently found and fundamentally motivational model component in the context of motivation models (4–6) and requires knowledge about the motives and image, before a potential fit can be discussed. This is displayed in Figure 2.

In sport psychology motives have been discussed for several decades and can be defined in terms of “… an affective-cognitive network of experiences, action alternatives, goals, and possibilities of need satisfaction that forms around a specific psychological need from early childhood on” (7). The decisive factor is—despite a certain stability—that a motive is subject to change within the framework of dynamic (transactional) processes: “These networks grow over time through experiences of need satisfaction or frustration” (7). According to this initial description, participation in handball clubs only occurs if children and young people can either associate “playing handball in a club” with previous positive experiences (e.g., previous satisfaction of needs or achievement of goals) or anticipate such positive experiences based on available information, e.g., experiences of third parties or their own experiences that are subjectively similar to playing handball. Positive experiences are determined within motives in particular by the fact that the satisfaction of basic psychological needs or the achievement of goals, purposes, and meanings is remembered or anticipated.

Our last description suggests an interdisciplinary perspective because in sport pedagogy Kurz (8) has reflected in particular on the question of the meaning that children and young people associate with “their” sport and exercise activities. Thus, the question of what meaning people see in sporting activities is ultimately linked to motives (9) or motive complexes (8) and the attitudes or expectations associated with them (10). In this regard, attempts have repeatedly been made to organize individual attributions of meaning to athletic activities into a manageable system (10). Finally, this has led to the following classification of meanings, which is particularly widespread in German-speaking countries, especially in documents about Physical Education (11–13): performance, excitement, social interaction, fitness and health, experiences with the body (impression), and quality of expression. These attributions of meanings to sport activities can be described in greater detail as follows:

– Practicing sport with a focus on *performance* means seeking out sporting situations because you expect to be good at them (presentation), to be able to show off in front of others (self-expression) or to prove yourself to yourself (self-affirmation). This orientation remains a central pillar of organized sport, but there are signs of a gradual decline (14).
– Practicing sports because you want to experience *excitement* means seeking out situations whose outcome is characterized by uncertainty. This applies in particular to games and competitions [e.g., (15)], but also to risk and adventure sports.
– *Social contact* as a motive for sporting activities motivates actions that aim to seek out opportunities to be with other people, experience sociability and communication, and process experiences while being together. For schoolchildren in particular, this seems to be a central reason for participating in sports in clubs with friends and acquaintances (16).
– The focus on *fitness and health*, which includes constructs such as balance, compensation, and well-being, must be considered specifically for children and adolescents in competitive sports, as the motive of participating in sports to be fit clearly dominates among them (17).
– Sport with the aim of experiencing one's own body (*impression*), exploring or seeking sensations implies emotions such as fun, flow or enjoyment of movement (18) and is also possible in traditional sports.
– The sense of *expression*, which includes synonyms such as aesthetics, design, presentation, or expression, justifies playing sports with the desire or goal of improvising, designing movements, or optimizing movement patterns, which ultimately brings the component of “playing beautifully” or “artistic value” into the discussion. Currently, this openness seems to appeal to many young people (19).

Table 2 summarizes examples of potential reasons for participating in handball due to these attributions of meaning.

**Table 2:** Attributions of meaning for sport participation in general and in handball.

Attribution of meaning	Children participate in handball activities because …
Performance	… they expect to be good at them (presentation), to be able to show off in front of others (self-expression) or to prove yourself to yourself (self-affirmation).
Excitement	… they seek out situations whose outcome is characterized by uncertainty.
Social interaction	… they to seek out opportunities to be with other people, enjoy socializing and communication, and process experiences while being together.
Fitness & health	… they want to be fit, athletically capable, and have a good-looking body.
Body impressions	… they imply emotions such as fun, flow, or enjoyment of movement.
Expression	… want to improvise, design movements, or optimize movement patterns, which ultimately brings the component of “playing beautifully” or “artistic value” into the discussion.

Our project aimed at describing and empirically exploring the decision for or against club-based sports activities as a result of a (lack of) fit between motivation and attribution of meaning as well as image. In this fit process, image was chosen as a motivational concept because it offers the most conceptual freedom to be associated with the complex concept of motivation. In recent (sports science) literature, the concept of image can be found in the field of marketing research, among others. Based on the definitions described above, images are defined here as the sum of attitudes or impressions of consumers (or addressees) with regard to a specific object (e.g., an organization or an event) (20, 21). Such images contain both cognitive and affective information (21). In sports-related images, this forms typical ideas or evaluations of sports activities, such as health, entertainment, exhaustion, exertion, or competition, which, depending on their intensity, are associated with different levels of sports participation (22). With regard to the development of images, most of the authors mentioned above assume that images are formed on the basis of experiences within person-environment processes. These experiences can be personal experiences or based on the experiences of third parties or one's own reflections [i.e., constructed experiences; cf. (23)]. In terms of sports, experiences can relate to environmental factors (e.g., training area, equipment, other players) or task factors (e.g., characteristic requirements, game dynamics, game hazards). If the image is based on information from third parties, this information comes from both personally significant others, known as “significant others” (24), and from social media or similar sources. In the case of significant others, depending on age, immediate family members (parents, siblings), friends, and other multipliers (e.g., teachers) are relevant for the formation of the image.

Summarizing these approaches, access to handball and thus to the (handball) sports club is likely, if the subjective image of the context of handball, or the entirety of all perceptions, ideas, and evaluations of the sport of handball, suggests that it satisfies individual needs or fulfills individual meanings, purposes, or goals. In this case, there is a fit between the individual motive and the subjective image of the sport of handball (motive-image fit). At the same time, the context of handball or the situational anticipations associated with it are attributed with the satisfaction of needs or a sense of purpose, or the fulfillment of goals and objectives is assumed [cf., also contextual offers or possibilities (3)].

## Method

### The MIPHa project

As part of the research project “Motivation-Image Fit in the Context of Handball” (MIPHa) conducted by the German Sport University Cologne and the University of Education Weingarten in cooperation with the German Handball Federation, we are pursuing the overarching goal of analyzing the mechanisms of sports club participation among children and young people in order to derive recommendations for action to recruit and retain members for handball. Specifically, children (aged 8–12) and young people (aged 13–14 and 17–18) are asked about their motivations for participating in and dropping out in sports in general and their image of handball. An exploratory sequential mixed-methods approach (25) was chosen as the research design, with research first conducted using a qualitative paradigm and then a quantitative paradigm. The following section focuses solely on the results of the exploratory qualitative study and on the subsample of children; it was conducted by the Weingarten research team.

### Research questions

(1) What motives are relevant for them to play handball?
(2) What are the main reasons why children end their handball careers?
(3) What image do children have of handball as a sport?

### Sampling

Once the ethics committee in Cologne had approved the project, sampling was carried out. The sampling process took place on two levels: at the regional level, represented by schools and clubs in various handball districts, and at the individual level of the participants. At the regional level, four different handball districts in Baden-Württemberg were selected (Oberschwaben-Ostalb, Bodensee-Neckar, Neckar-Franken, Neckar-Alb), and care was taken to select locations with and without a focus on handball. Access was provided by teachers and club coaches, who acted as gatekeepers. At the individual level, the selection of project participants followed the principles of theoretical sampling. Children from all three phases of club participation (active, no longer active, and inactive handball players) were contacted by the gatekeepers. This resulted in a sample of 35 participants. Pre-selection could not be ruled out, as the responsible teachers and coaches already knew the children and may have made a selection based on certain criteria (26). Table 3 provides an overview of the sample.

**Table 3:** Relevant features of our sample (children, 8–12 years). .

Gender	Active	No longer active	inactive	overall
*Male*	*5*	*3*	*8*	*16*
*Female*	*10*	*2*	*7*	*19*

### Data collection

The interview guide was developed by the entire project group. The interview concept was subjected to a pilot phase of testing, after which adjustments were made in accordance with the results. Before participants were recruited, they and their legal guardians received an informational letter and a consent form stating that they had been informed of the purpose and content of the survey and were required to agree to it. Following the receipt of consent forms from legal guardians, the interviews were conducted between October 2024 and February 2025 with the participants in a school setting during or after lessons, or in a club setting before, during, or after training (approx. 30–45 min.). With the participants' consent, the interviews were recorded using a recording device. Data collection took the form of individual interviews, which were conducted in parallel by two members of the research team in face-to-face conversations with a single respondent. This method of data collection was chosen because it allows opinions and impressions to be expressed freely and directly. Childhood research has shown that open methods, in which the children being interviewed can largely control the interview situation themselves, are best suited to the requirements of qualitative research with children (27). The researchers noted any relevant observations, statements, or movements. Semi-standardized (guide-based) interviews were used, which, in addition to their usual features (including open-ended questions and narrative prompts), took into account the age of the target groups in particular through child-friendly wording and visual stimuli (28). The interviews included questions about reasons for and against handball (motivation analysis), the image of handball players, and the sport of handball (image analysis).

### Data analysis

After the interviews had been transcribed verbatim, the texts were completely anonymized and analyzed by two members of the research team using qualitative content analysis (29, 30) to identify motives and image statements with the MAXQDA 2024 program. In a first step of the analysis, the categories and subcategories were derived inductively from the empirical material. In a second step, the texts were then analyzed using deductive content analysis with regard to theoretical classification (goal orientation, needs, attribution of meaning).

## Results

### Inductive qualitative content analysis: reasons for and against handball

The participants' statements about their reasons for and against handball clubs can be divided into six main subject areas which encompass social, performance-related, sport-specific, physical, emotional, and structural aspects. An overview of these categories and their respective specific characteristics are presented in Table 4.

**Table 4:** Categories and key reasons for and against handball.

Category	Key reasons
Social aspects	**in favor:** friends, team, coach, family
	**against:** dissatisfaction with coach/team, lack of fairness
Performance-related aspects	**in favor:** competence, competition, sense of achievement, striving for performance
	**against:** failure, experience of incompetence, lack of opportunity to play, pressure to perform
Sport-specific aspects	**in favor:** enthusiasm for sports activities, interest in sports, characteristics of sports, school-based introduction
	**against:** complex set of rules, lack of understanding, poor school-based introduction
Physical aspects	**in favor:** staying fit, working out, physical balance, physical challenge
	**against:** physical strain (risk of injury, pain), foul play
Emotional aspects	**in favor:** excitement and suspense, mental relaxation
	**against:** fear, frustration, lack of motivation
Structural aspects	**in favor:** proximity to home, positive club features, regularity/routine, training
	**against:** dissatisfaction with training/playing, time constraints, lack of opportunities, financial burden

Regardless of age and gender, the social context plays a major role. Many children report that they participate in sports primarily because of the friendships and sense of community within the team. They talk about how they have made new friends in the club and how much the team spirit and camaraderie motivate them. Team spirit, mutual support, and shared successes are particularly important to them. Contact with like-minded people and exchanges with coaches and teammates are also seen as very enriching.

“The community. Being together and sticking together. (..) Rejoicing together when you win”. (NMH2, Pos. 44).

The inductive analysis also demonstrates that another influencing factor is the family environment: many children were introduced to handball by their parents, siblings, or relatives and became enthusiastic about it.

In addition to these social aspects, the desire to perform well also plays an important role. Many children report that they find it particularly exciting to continuously improve in training and in games. They want to learn new things—such as new throwing techniques or tactical behavior in the game—and are happy when they make noticeable progress. The feeling of having achieved something gives them self-confidence and is particularly enjoyable for them. Teamwork—supporting each other and working together to score goals—is also a key reason.

“It's fun, you get better, you meet new kids—and you just have a good time”. (H3, Pos. 109–110).

Another aspect that matters is the recognition they receive for good performance—for example, praise from the coach or a nomination for selection teams. Overall, the picture is positive: for many children, the experience of competence, i.e., noticeable progress, a sense of achievement, and positive feedback from those around them, is a key reason for playing handball. Some children also report long-term goals: they want to move up to higher youth teams, play for famous Bundesliga clubs, or even make it into the national team. Within this context, the desire for recognition and visibility also plays a role—for example, appearing on television or being allowed to play in major tournaments.

A further notion that is often mentioned is enthusiasm for the sport itself. The children emphasize how much they enjoy the game itself—scoring goals, throwing the ball, doing new exercises. Above all, they appreciate the variety, the versatility of the movements, and the opportunity to constantly try new things as part of the team. Physical aspects such as health or fitness are mentioned much less frequently, but are still present: some children say that they enjoy exercise because it is good for them, fun, and they don't want to sit around all day.

School, school sports, and physical education plays an ambivalent role: on the one hand, for many children it is their first contact with handball—for example through extracurricular activities or action days like the “Grundschul-Aktionstag”—and can spark enthusiasm. On the other hand, many children report feeling overwhelmed, having inadequate equipment, or experiencing a lack of fairness in class.

“I don't think the balls at school are any good. And nobody can play it at school”. (H4, Pos. 198)

Going one step further, we conducted a between-group analysis comparing children who play handball (group 1), those who are not playing anymore (group 2), and kids who have never played handball (group 3). In fact, we were able to identify some interesting differences:

1. Starting with the first group, it has been shown that their focus is on performance-related aspects—in particular, the experience of competence and the pursuit of improvement and progress.
  “ …. that the training is usually varied. And because I'm learning a lot of new things and making good progress”. (H9, Pos. 67–68)

For those kids it is particularly motivating that they develop further, learn new things and experience progress in the matches.

2. Children of group 2 emphasize on the other hand social aspects. They particularly enjoy reminiscing about the sense of community within the team and playing sports together with friends. One child describes this as follows:
  “Terms that are important to me in handball are friendship, teamwork, and fun” (NMH5, Pos. 88). This shows that even if they no longer participate in the sport themselves, it is the social experiences that remain particularly positive memories.
3. Finally, children who have never played handball in a club themselves, but who are familiar with it mainly from school sports and also from the media. These children show a different perspective—they report a fascination with the sport itself. One child describes it this way:
  “It was a lot of fun. Especially how precisely you have to play and how you score the goal”. (NH4, Pos. 63–64)

This means that their focus is primarily on the playful enthusiasm for handball—the game, the technique, the goal-oriented nature.

Overall, the differentiation shows that while active players talk a lot about performance motivation, former active players mainly look back on the social interaction. Children with no club experience, on the other hand, perceive handball primarily as an attractive, versatile sport—often influenced by the school context and, in some cases, by television.

In addition to the numerous motivating factors, children also mention stressful issues that discourage them from playing handball. Respondents report physical stress—including acute pain from injuries, general physical exertion, but also the risk of injury, which is sometimes perceived as a deterrent. Social aspects also play an important role. Some children describe dissatisfaction with the team or the coaches, reporting unfair treatment, a lack of fairness, or unpleasant interactions.

“If you don't fit in, I usually don't think that's cool. (…) Then you just stand around. And that feels kind of stupid”. (NH7, Pos. 109–112)

It is striking that children are more likely than adolescents to talk about performance-related reasons: many find failure—such as losing games—frustrating. Several report feeling disappointed, withdrawn, and losing their enjoyment of the game. Some children feel pressure to perform, are afraid of making mistakes, or feel ashamed when they compare themselves to better players. In such moments, they feel incompetent or like they don't belong. Another key aspect is time constraints: many children feel a lot of pressure from school, homework, or other obligations at a young age, as well as often pursuing several hobbies at the same time. This can lead to training sessions being skipped or even giving up the sport altogether. Overall, it is clear that the decision to give up handball is rarely one-dimensional. It is usually the result of a combination of several factors

At this point, we would like to take another look at the three activity groups with regard to reasons for being critical of handball or even decide against it:

1. Focusing children of group 1 we could state that they also have critical arguments, in particular with regard to training and game strategy. One key issue is dissatisfaction with playing time—both in training and in competition. One child sums it up very clearly:
  “I would prefer to eliminate the obstacle course and just play handball, because then we could actually play handball for longer”. (H6, Pos. 78)

In addition to wishing more playing time, children also criticize one-sided training content, the behavior of individual teammates, the strictness of coaches, and perceived unequal treatment—such as preferential treatment of selected players. Overall, it seems to be clear that children want more co-determination, more varied, game-oriented training, and supportive, fair cooperation.

2. Regarding children of group 2 it is striking that physical aspects are frequently mentioned. One child, for example, reports that he stopped playing handball due to injuries—specifically a dislocated arm—and now prefers to play soccer. The sport handball is perceived as too rough and risky, which caused anxiety. Other children cite external circumstances such as moving to another place or a lack of clubs in their new place of residence as reasons for giving up handball.
3. Finally, we describe the attitudes of those children who belong to group 3. These kids mention various reasons why they tend to reject handball or find it difficult. The reasons include negative experiences (mostly in physical education classes), for example due to injuries, fear of the ball, or physically dominant behavior by classmates. The complexity of the rules is also often perceived as an obstacle—in particular, traveling and fouls seem difficult for many to understand. Additionally, some children report feeling overwhelmed—for example, when throwing or catching the ball—which leads to a feeling of incompetence and reduces the enjoyment of the game.
  “And then there's all the back and forth. If you miss a ball, the others yell at you for missing it. Even though it was a good play, just because you didn't see it”. (NH1, Pos. 108)

Overall, our inductive analysis has shown that the reasons against handball are complex: They range from organizational and structural aspects to social tensions as well as physical and emotional stress; and they vary greatly depending on the group and individual experience.

### Deductive content analysis and theoretical classification

The deductive analysis of the participants' statements according to Kurz's categories of meaning illustrates how multifaceted children's experience and interpretation of handball is; Table 5 gives an overview of our analyses.

**Table 5:** Attributions of meaning and key quotes.

Attributions of meaning (Kurz,1977)	Key quotes from the qualitative study
Performance	“I think it's great that people try to do their best”. (H2, Pos. 27) “I think it's really special when my coach praises me during a handball game and tells me I've done something well. Or when I've achieved something that I really didn't think I would achieve, or that I thought would be relatively difficult to achieve”. (H13, Pos. 20)
Expression	“When I took the seven-meter shot and scored. I really enjoyed that. Or generally when I was mentioned in the press release because I scored the most goals. It's just a great moment”. (H4, Pos. 52) “Where I then scored a lot of wild goals a few times in a row and was able to show what I can do”. (NH7, Pos. 114)
Impression	“I really enjoy the exercises because they are always different”. (H8, Pos. 63–64) “I would like to try handball because I really enjoyed it in PE at school”. (NH3, Pos. 94)
Fitness and health	“I just think it's cool. Exercise and stay fit. Don't just sit around”. (NMH, Pos. 34) “I exercise because I want to be fit and healthy”. (H11, Pos. 12)
Social interaction	“I actually get particular joy from simply winning games with my friends”. (NH7, Pos. 22) “I like teamwork when it's good, when everyone gets along well”. (NH4, Pos. 44)
Excitement	“The game, you're kind of excited, you're happy. And the excitement at the beginning”. (H10, Pos. 142) “It is exciting to try out new things in sports”. (H8, Pos. 12)

The category of “performance” shows that many children associate their involvement in sport with the desire for further development and recognition. Their own progress, team success, and the prospect of being selected for teams give the sport a purposeful meaning. As to the issue of “expression”, it becomes clear that children also experience handball as an opportunity to show themselves creatively and individually—for example, through spectacular throws such as the Kempa or specific game situations that allow them to stand out from others and bring their personality into play. The meaning “impression” reflects the perception of the sport: while some children find training varied and fun, others—especially in a school context—report frustration and a lack of enthusiasm. The experiences here are highly context-dependent. The idea of “fitness and health” is also present: many children describe exercise as important for their own well-being. They want to stay active, feel healthy, and prevent negative physical developments such as obesity. “Social interaction” is a central theme that runs through almost all of the statements. Social cohesion, shared experiences, and the importance of team spirit have a significant influence on many children's perception of handball and give the sport a unifying function. Finally, the theme of “excitement” shows that children find this fast-paced, dynamic game particularly thrilling. Competition, excitement, and emotional highs and lows make handball an exciting experience for many, inspiring and motivating them.

### Image of children playing handball

When we asked children what comes to their minds when they hear the word “handball”, we got a very diverse picture—but with clear focal points. It's about playing the ball with your hands, throwing it accurately, dribbling, scoring goals—in other words, technique, coordination, and precision.

“We learn handball in physical education class. And that's where I always see these examples. For example: We train with a ball and we have to throw it into the goal. We have to dribble, we have to jump”. (NH4, Pos. 104)

Children associate it with a dynamic, active game in which physical and technical skills are important. In addition, handball is often described as a team-oriented sport. Many children emphasize the importance of teamwork, team spirit, and fairness. For some, handball is also a unifying sport in which you can quickly make new friends and have fun. Some children also describe handball as an exciting sport that looks cool and it's fun to play.

“Well, I've watched handball and I think it always looks really fun, the way they… (…) run and catch the ball and stuff, it looks really cool”. (NH2, Pos. 126–127)

Idols also play a role. Children who play handball in particular report admiring well-known professionals. On the other hand, there are also children—especially those who have no contact with club handball—who have little idea about the sport. Others report that they imagined the sport to be less physical or fairer. The impressions reflect the children's diverse images of handball as a sport. It is striking that active children emphasize above all the joy of improvement and competition in relation to handball, while respondents who are no longer active remember the social interaction, and children who do not actively play handball perceive the sport as technically demanding, physically challenging, and incomprehensible. The associations differ significantly depending on the children's own experiences. For children who actively participate in handball, as well as former players, the image is largely shaped by their own practical experiences, which have a lasting influence on their perception of the sport. Non-active children, on the other hand, derive their image of handball primarily from their social environment. Stories from friends, acquaintances, or family members who are or were active themselves are often mentioned. Teachers in a school context also contribute to shaping the image of handball. Digital media such as Instagram or TikTok play a minor role in this context. Instead, children more often report analog sources of information such as posters or flyers at school, presentations by club staff, or specific handball action days. In individual cases, watching international tournaments such as World or European Championships on television is also mentioned as a formative element.

### Initial findings on the motive-image fit

Motive-image fit describes the interplay between the individual motivations of children and young people (motives) and their subjective perception of the sport (image). A fit exists when the image of handball matches the personal expectations, needs, or goals of young athletes. Only in this case does a lasting bond with the sport and the club develop.

Our study shows that children and young people only turn to handball or remain loyal to it if they perceive the image of the sport as meaningful and motivating. This means that they perceive handball as a sport that fulfills their personal goals—such as fun, community, success, or physical challenge. If this fit is missing, for example, if the image of handball is perceived as too competitive or physically demanding, even though the individual's motivation is more focused on playful movement and social closeness, they will turn away from the sport.

The first motive-image fit in our study we could find out is the following connection: Children who initially experienced handball as a unifying and team-oriented sport can only identify with the sport in the long term if this image remains intact and corresponds to their personal motives—such as belonging, enjoyment of exercise, or recognition. If the image changes or the fit is no longer there, the likelihood that the child will end their sporting career increases.

**Figure 3:**
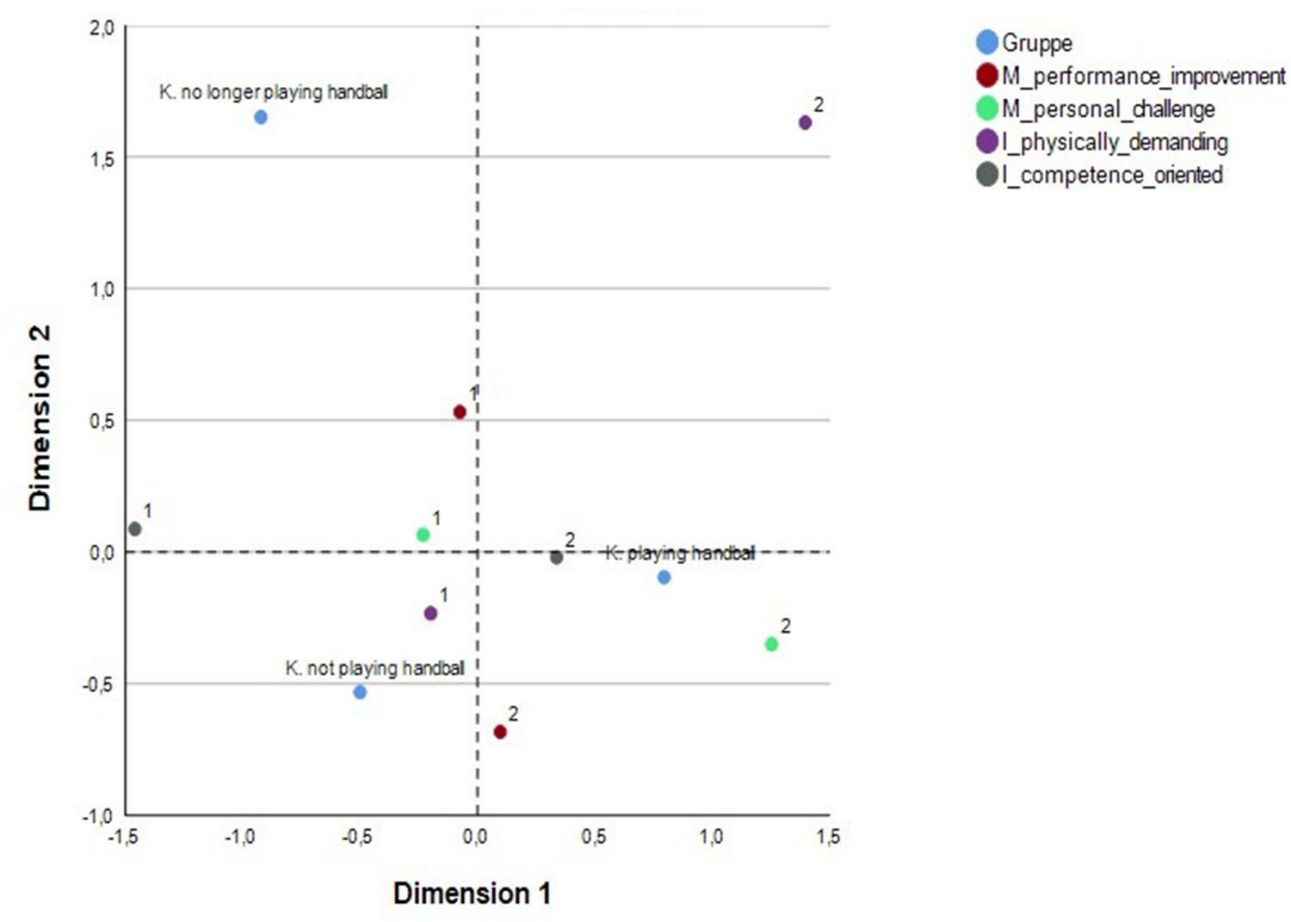
Correspondence analysis of the motive-image fit of performance-related aspects (K = kids).

Correspondence analysis could be a fruitful approach for a more precise analysis of motive-image matches on a quantitative level. This statistical procedure is considered as relatively low-threshold, as it does not place any major demands on the variables and is applicable for large contingency tables (31). The only requirement in this case was data transformation which we implemented with the dichotomy of “present” (1) vs. “non-present” (32, 33) to explore some specific fits. Here, “present” means that a participant mentioned this category, “non-present” stands for the opposite, assuming that mentioning a category pointing out a certain significance of this category. A correspondence analysis then distills categorical data into a simple visual map. It uncovers patterns by showing how rows and columns of a contingency table relate to each other in a low-dimensional space. The method highlights which categories behave similarly and which differ, making hidden structures in the data easier to interpret (31). Figure 3 gives an example of the motive-image fit of performance-related aspects.

As can be seen, we could explore a motive-image fit for children because the motives “personal challenge” and “performance improvement” as well as the image “handball as competence-oriented sport” are relatively close by. However, this highlighting of a hidden structure in our data is just an exploratory beginning (work in progress) and needs further enhancement in order to be recognized as a robust database.

## Discussion

Our analyses brought various issues to light: First, the social context including the aspect of teamwork plays a significant role with regard to children's motives. In addition, the concepts of performance, exercise, and competition contribute to children getting excited about handball. Finally, we were able to develop the idea that children also seek the limelight of public attention. Discussing these issues with reference to our initial comments on the concept of sustainability of a sport, we want to argue as follows:

The issue *social context* shows that it is important for children that their individual sports activities make sense, when friendships are formed and strengthened. Team spirit plays a central role for them (34) and contributes to long-term commitment to a sport. Feeling comfortable and socially integrated aligns with the theoretical dimension of belonging (35). The opportunity to train, celebrate, and also “fight” together creates emotional bonds that go beyond pure physical activity. This social component is an essential building block for the sustainability of a sport, as it strengthens motivation beyond pure performance goals and ensures long-term member loyalty (34, 36). A club that actively promotes this social aspect—for example, through team events, joint excursions, or a strong club culture—creates an environment in which children feel “at home” and want to stay; they feel comfortable (11).

With regard to the ideas of *performance, exercise, and competition* children obviously regard handball as a dynamic and active sport. Exciting games, in which they give their best and are rewarded with success, leave many with positive memories. The combination of fast play, technical skills, and tactical thinking promotes both physical and mental development (37). This aspect of performance and competition is a key incentive for children to choose handball, whereby this takes place on both the level of self-reference and in relation to peers (38). The opportunity to surpass themselves, learn new skills, and succeed in a team strengthens their self-confidence and motivation (39). In terms of sustainability, this underscores the importance of clear performance structures and visible developmental pathways. When children see that their efforts are rewarded, e.g., by winning competitions (39), their loyalty to the club and the sport increases.

Finally, and with a focus of seeking the *limelight of public attention* the participants of our explorative study often showed future-oriented plans. They emulate their role models from the national teams and would like to play there one day (40). Many children also report that they became aware of the sport through posters, flyers, and club promotions, as well as through the Handball World Cup and European Championships on public television or through friends, acquaintances, or family members. This aspect of the “limelight” is a decisive factor in the appeal of handball. The opportunity to be in the spotlight, receive recognition, and serve as a role model is a strong motivator for children (39, 41). This shows that public perception and the image of a sport are crucial for recruiting members. Club that strengthens the image of handball through a strong public presence, the promotion of talent, participation in regional and national competitions, and the use of social media—can arouse children's curiosity and ambition (42).

In sum, it can be said that the sustainability of handball does not depend solely on the quality of training opportunities or the number of games, but on a complex interplay of social, performance-related, and image-related factors. Social bonding, enjoyment of the game and competition, as well as the opportunity to be in the spotlight, are the central pillars that motivate the children in our study to choose a sport and stick with it in the long term. A club that actively promotes these dimensions—a strong team spirit, a clear performance structure, and outstanding public relations—creates the basis for sustainable membership recruitment and a vibrant club culture. The future of handball does not only lie in developing talents, but also in creating an attractive and welcoming environment where children can feel comfortable, grow, and realize their dreams.

## Conclusion

Based on a development of membership and team numbers in the German Handball Federation this article dealt with adolescents of 8 to 12 years, their motives for playing or not playing handball as well as the image they have about handball. Before this background we wanted to find out, if the motives of our target group and their subjective perception of this sport (image) fit. The corresponding research questions were addressed with an exploratory sequential mixed methods design, from which we reported the first and qualitative strand. Overall, we found out that children will only take up handball on a long-term and sustainable basis if their individual needs, goals, and the aspects of the sport that are meaningful to them are recognized and taken into account. To ensure that handball remains attractive to children, beneficial factors such as community, enjoyment of the game, experiencing competence, and opportunities for self-expression should be specifically reinforced. At the same time, barriers such as social tensions, pressure to perform, or structural hurdles must be minimized. Only through a sensitive balance of motivation, recognition, and positive experiences can handball offer children's meaningful prospects—and thus contribute to the future viability of the sport.

However, the results and the recommendations yet to be formulated must also be viewed from the perspective of potential limitations of the study. We must be aware that a possible generalization of the results might be biased by the sampling process because our all participants came from the South of Germany. Additionally, we should re-examine our interview guideline as it might contain inadequate questions. Finally, interpretative data analysis could also be misleading due to researchers' bias. Thus, we urgently studies replicating our approach to get more robust data.

Nevertheless, our results ultimately enable us to make a number of recommendations to various stakeholders:

– *Targeted public relations work:* Flyers, posters, and club activities such as taster courses or open training sessions should be used by clubs to introduce children to handball at an early age and in a low-threshold manner.
– *Involve multipliers:* Families, friends, acquaintances, and teachers should be used as important intermediaries. Information materials and invitations to club taster days can be distributed via these channels. This requires coordinated action by the clubs.
– *Emphasize positive motives:* Communication and training offerings should focus on social aspects, diverse forms of exercise and play, and fun to increase the appeal of the sport, which is a future challenge for coaches' training.
– *Reduce pressure to perform:* Simplified rules and forms of play should be offered, especially for children with no previous experience, in order to reduce inhibitions and make it easier to get started. Thus, coaches' behavior should be guided rather by improving the individual performance and a certain distance to solely aim for peak performance.
– *Expand media presence:* Greater visibility of handball on social media and television can improve the image of the sport and spark children's curiosity. Simultaneously, handball clubs should maintain a regular presence on social media and local newspapers to give their players a certain publicity.
– *Take advantage of major events:* World and European championships offer ideal opportunities to carry out accompanying youth activities and get children excited about handball, a recommendation in particular for the national association and potential marketing activities
– *Expand school offerings:* Action days, teaching materials, and attractive teaching formats for schools should be intensified in order to integrate handball more strongly into everyday school life. In this context, the bridge between school and clubs should be *strengthened:* The integration of handball into all-day programs creates a direct link between school sports and club structures.
– *Communicate unique selling points:* In view of competition from other sports such as basketball and volleyball, handball should clearly highlight and proactively communicate its particular strengths—team spirit, dynamism, and excitement. However, this should not be limited to the umbrella organization, but in particular on the local level realized by club initiatives.

Children must not only be won over, but also retained in the long term. This requires continuous support, attractive club offerings, and close cooperation with schools and families. In the long term, the DHB can increase the appeal of handball for children, attract new members, and retain existing ones in the long term through a dual strategy of image promotion and barrier removal. In doing so, it is making a decisive contribution to the sustainable development of children's handball and securing the future of the sport in Germany.

## Data Availability

The raw data supporting the conclusions of this article will be made available by the corresponding author, without undue reservation.
